# Perceptions of HIV cure and willingness to participate in HIV cure-related trials among people enrolled in the Netherlands cohort study on acute HIV infection

**DOI:** 10.1016/j.jve.2022.100072

**Published:** 2022-06-15

**Authors:** Pien van Paassen, Maartje Dijkstra, Holly L. Peay, Casper Rokx, Annelies Verbon, Peter Reiss, Jan M. Prins, Gail E. Henderson, Stuart Rennie, Pythia T. Nieuwkerk, Godelieve J. de Bree

**Affiliations:** a Department of Internal Medicine, Division of Infectious Diseases, Amsterdam UMC, Academic Medical Center, Amsterdam, the Netherlands; b Department of Infectious Diseases, Public Health Service of Amsterdam, Amsterdam, the Netherlands; c Department of Social Medicine, University of North Carolina-Chapel Hill, USA; d UNC Bioethics Center, University of North Carolina-Chapel Hill, USA; e RTI International, Research Triangle Park, NC, USA; f Department of Medical Psychology, Amsterdam UMC, Academic Medical Center, Amsterdam Public Health Research Institute, Amsterdam, the Netherlands; g Department of Internal Medicine and Department of Medical Microbiology and Infectious Diseases, Erasmus University Medical Center, Rotterdam, the Netherlands; h Department of Internal Medicine, Division of Infectious Diseases, University Medical Center Utrecht, Utrecht, the Netherlands; i HIV Monitoring Foundation, Amsterdam, Noord-Holland, the Netherlands; j Department of Global Health, Amsterdam Institute for Global Health and Development, Amsterdam UMC, University of Amsterdam, Amsterdam, the Netherlands

**Keywords:** HIV cure, Acute HIV infection, Analytical treatment interruption, In-depth interviews, Perceptions

## Abstract

**Background:**

People who initiate antiretroviral therapy (ART) during acute HIV infection are potential candidates for HIV cure-related clinical trials, as early ART reduces the size of the HIV reservoir. These trials, which may include ART interruption (ATI), might involve potential risks. We explored knowledge and perception of HIV cure and willingness to participate in cure-related trials among participants of the Netherlands Cohort Study on Acute HIV infection (NOVA study), who started antiretroviral therapy immediately after diagnosis of acute HIV infection.

**Methods:**

We conducted 20 in-depth qualitative interviews with NOVA study participants between October–December 2018. Data were analyzed thematically, using inductive and iterative coding techniques.

**Findings:**

Most participants had limited knowledge of HIV cure and understood HIV cure as complete eradication of HIV from their bodies. HIV cure was considered important to most participants, mostly due to the stigma surrounding HIV. More than half would consider undergoing brief ATI during trial participation, but only one person considered extended ATI. Viral rebound and increased infectiousness during ATI were perceived as large concerns. Participants remained hopeful of being cured during trial participation, even though they were informed that no personal medical benefit was to be expected.

**Interpretation:**

Our results highlight the need for thorough informed consent procedures with assessment of comprehension and exploration of personal motives prior to enrollment in cure-related trials. Researchers might need to moderate their expectations about how many participants will enroll in a trial with extended ATI.

## 1 Introduction

The field of HIV cure-related research is challenging and rapidly evolving.^1^ Current studies vary in design and focus on gaining knowledge of how to achieve a “cure”, understood as either complete eradication of replication-competent virus (‘sterilizing cure’) or as reaching a durable control of HIV in the absence of antiretroviral therapy (ART) (‘functional cure’, ‘post-treatment control’ or ‘remission’).^2^ Key targets for HIV cure-related research for the next five years are recently described by the International AIDS Society (IAS).^3^ A significant obstacle in curing HIV is the presence of a latent viral reservoir. Several researchers have attempted to understand potential sources of viral rebound, and concluded that HIV can persist in numerous forms, cells and tissue locations. Reaching HIV remission will very likely consist of a treatment that targets both the viral reservoir and host immunity.^4^ Previous studies have shown that starting ART in the acute phase of infection limits the formation of the viral reservoir and could possibly preserve the immune system.^5^ People with potentially smaller viral reservoirs as a result of starting ART early after infection are potential candidates for trials aimed at curing HIV.^6^ To investigate cure interventions, analytical treatment interruption (ATI) is needed because until today no biomarker has been identified to predict viral rebound.^4^

Participation in trials involving ATI may pose a burden on study participants, because both reinfection with drug resistant HIV could occur^7^ and viral rebound after ATI results in an increased likelihood of transmitting HIV to sexual partners.^8^^,^^9^ Also, participants are temporarily deprived of the physical and mental therapeutic benefits of ART (10, 11). Moreover, trials with ATI often include intensive and invasive tissue and blood sampling^12^ and it is unlikely that participants will have personal medical benefits from the study intervention.^10^ This could impact their willingness to participate in these HIV cure-related trials and is critical in the design of cure-related trials.

In this context, a recent study from Belgium showed that for most participants in a study in which participants started ART in the acute phase of infection and underwent ATI the contribution to a possible cure outweighed the burdens and risks of participation.^13^ This is in line with qualitative findings from a cure-related trial (including ATI) in Thailand.^14^ Interviews conducted in the latter study revealed a discrepancy between what researchers imagined to be the benefits of joining a cure-related trial, and what participants actually perceived to make participation worthwhile. For example, some participants felt this gave them a status of being ‘special’ contributors to scientific knowledge. These findings suggest that motivations driving trial participation appear to be more complex than what experts in research ethics may expect.^14^ However, these studies have been conducted among participants who were already participating in an ATI study and may have therefore selected people with positive attitudes towards ATI. Furthermore, as stated by the IAS, there is an increased need in putting ethical, social and behavioral considerations regarding HIV cure-related trial participation on the scientific agenda as it affects the feasibility of future research and the well-being of participants.^4^ To gain insight in motivations and barriers of potential participants of cure-related trials, as well as related challenges with informed consent, it is important to gather data in different social and research contexts.^15^^,^^16^

Therefore, we explored perceptions towards HIV cure-related trials among participants of the Netherlands Cohort Study on Acute HIV infection (NOVA) study. The NOVA study is a multicenter observational prospective cohort study in the Netherlands enrolling participants with acute HIV infection starting ART immediately upon diagnosis.^17^ This paper focuses on perceptions of participants regarding their knowledge and understanding of HIV cure, their perception of the relationship between being diagnosed with acute HIV infection and cure, and drivers and barriers to participate in HIV cure-related trials.

## 2 Methods

The in- and exclusion criteria for the NOVA trial, and details of diagnostics and antiretroviral therapy have been reported elsewhere.^17^ Participants in active follow-up at the Amsterdam University Medical Center (AMC site) were invited for an in-depth interview. Participants were informed about the study by phone and with a leaflet describing this sub-study and its objectives. Informed consent was given verbally and audio-recorded before the start of the interview.

### 2.1 Data collection

In-depth interviews were conducted between October–December 2018. The interviews were performed by two researchers (MD and SR), who did not have any relationship with the participants prior to these interviews. MD received basic training in qualitative health research, including conducting in-depth interviews. SR had conducted in-depth interviews in several previous studies. Interviews were conducted both in Dutch and English, depending on participant preference. The interviews lasted approximately 2h and were performed in a private room at either the HIV clinic or the Medical Psychology department of the Amsterdam UMC. The interview guide was discussed after every three interviews and adjusted accordingly. The topics discussed focused not only on HIV cure, but also on experience with the diagnostic trajectory, immediate start of treatment, disclosure of participation in the NOVA study and their HIV status and partner notification. To explore the willingness to participate in cure-related trials, participants were presented two fictional (hypothetical) scenarios. Scenario 1 included brief ATI, scenario 2 extended ATI (see Box 1). Interviews were transcribed by a professional transcription company. Additional participants were interviewed until thematic saturation was attained. Thematic saturation was defined as no new themes emerging after three consecutive interviews.

Box 1 **Scenarios of cure-related trials**

Scenario 1.

• Participants would get several doses of an experimental drug.
• They would later stop their HIV treatment (ART).
• Participants would go to the hospital at least weekly for an exam and blood draws to monitor overall health, viral load, and CD4 count.
• The experimental drug would probably be safe, but researchers don't know for sure. Some participants would have mild side effects.
• During the time off HIV treatment (ART):
  o The virus would be detectable in the blood of most participants a few weeks after stopping their HIV treatment. With some participants, it will take longer before this happens.
o Most participants would not have any HIV symptoms.
o Participants may have an increased risk of transmitting HIV to partners.
• As soon as the virus is detectable in the blood, HIV treatment (ART) would be started again. The overall health of the participant would then be checked in the usual way at the hospital.

Scenario 2.

The **differences** with scenario 1 are highlighted in **bold**.

• Participants would get several doses of an experimental drug. **They would later stop their HIV treatment (ART) for a much longer time.**
• Participants would go to the hospital **every two weeks** for an exam and blood draws to monitor their overall health, viral load, and CD4 count.
  • The experimental drug would probably be safe, but researchers don't know for sure. Some participants would have mild side effects.
  • During the time off HIV treatment (ART):
o The virus would be detectable in the blood of most participants a few weeks after stopping their HIV treatment. With some participants, it will take longer before this happens. **After the virus is detectable in the blood, participants would continue to remain off their HIV treatment (ART) for about another month, depending on the health of the participant.**
o **Some participants might have mild HIV symptoms.**
o Participants would be at **high risk** of transmitting HIV to partners.
• As soon as HIV treatment (ART) is restarted again, the overall health of the participant would then be checked in the usual way at the hospital.

Alt-text: Box 1

### 2.2 Data analysis

MD, SR, PP and PTN conducted the coding. Important themes were identified in advance based on the research questions and findings from previous studies. We identified sub-themes and codes by using a mixed deductive/inductive and iterative approach. The first three interviews were coded by all four coders independently, who developed an initial coding tree. We revised this coding tree through discussion until a consensus was reached. The remaining 17 interviews were coded by two coders independently, who used a consensus approach for discrepancies in coding and who also discussed and revised the coding tree after coding. Codes were categorized into the pre-identified themes. The analysis was done using MAXQDA version 12.0.

### 2.3 Ethics of participation

The study was approved by the Medical Ethics Committee of the Amsterdam UMC (W18_183#18.222).

## 3 Results

At the time of participant selection, 34 people were in active follow-up in the NOVA cohort study at the Amsterdam UMC. Of these, seven people were not invited for the present study because they had recently participated in a different interview study (n = 4) or saturation was reached (n = 3). In total, 27 people were invited for participation in the present sub-study. Of these, seven declined and 20 people were enrolled. Table 1 depicts baseline characteristics of the participants. All twenty participants were men, with a median age of 38 (range 21–60) years. Eighteen participants were mostly or exclusively attracted to other men. Median time between starting ART and the interview was 20 (range 3–48) months.

**Table 1:** Baseline characteristics.

Qualitative sub-study participants' characteristics	
Number of participants	20
Male gender	20 (100%)
Median age (in years)	38 (range 21–60)
Median time between diagnosis and starting ART (days)	3 (range 0–25)
Median time between start ART and interview (months)	20 (range 3–48)
Country of birth	
The Netherlands	13 (65%)
Surinam	2 (10%)
Jamaica	1 (5%)
Denmark	1 (5%)
China	1 (5%)
Australia	1 (5%)
Highest level of education	
Primary school	0 (0%)
Secondary school	3 (15%)
(Higher) Vocational education	11 (0,55%)
University	6 (30%)
Sexual preference	
Predominantly men	13 (65%)
Men and women	6 (30%)
Predominantly women	1 (5%)

In Table 2, the themes regarding HIV cure are described, with corresponding sub-themes and quotes.

**Table 2:** Summary of themes, sub-themes and corresponding quotes from in-depth interviews about HIV cure-related trials among NOVA study participants.

Importance of HIV cure		
1. No more medication	Worry long-term use causes damage to the body	*I'm afraid of the consequences of taking ART for years and years. Before, ART was known to cause osteoporosis and you know the images from television of men with AIDS having sunken faces and a pale appearance. Very skinny, with a lot of weight loss, eventually dying from AIDS. […] I'm afraid that's waiting for me.*
	Burden of daily medication	*It would feel like a bit of weight falling off your shoulders. Then you wouldn't have to think about taking a pill every day. I mean, I would love to delete that alarm from my phone (laughs).*
	ART reminds them of diagnosis	*I was not really concerned with having to take medication every day, because I was only thinking about the fact that I was infected. And when you get these pills and you come to realize, not that I have to take this every day, but more that this is what your life will be.*
		*Taking medication every day is a reminder that you are sick.*
2. Impact on relationships	No longer living with a secret	*Not having to keep a secret, not having to rearrange things in my house when someone comes over, having to hide the pills. I think that is the worst part of it. For me, it would mean that I would be free again.*
		*It would be such a relief, especially socially. Just not having to … that you can live without a secret.*
	Starting a new relationship	*It would let me live a normal life again, that I don't have to consider with a girlfriend: oh, I have this, how will she respond?*
3. Becoming normal	Starting over	*Out of my body, yes. A second chance.*
		*A cure. Just the sound makes me excited. A first start, a start over, a second chance.*
	Normalization	*The sooner you're undetectable, the sooner you're normal again.*
		*It would mean that I can be normal again.*
4. Cure not so important	Already feels cured by ART	*It doesn't weigh on my mind that I have HIV. If I was to be given a magic tablet that would take it away, I'd probably feel the same as I do now.*
		*If I can be healthy and grow old with my medication, then the price I want to pay for cure is relatively low. The better my treatment is, the lower the price I'm willing to pay for cure.*
	Back to fear of contracting HIV when cured	*Even if, say, I were cured, I would still want to take PrEP. Then you go from 3 pills to 2 pills. That's not such a big difference. In fact, I would not want to stop antiretroviral therapy, because I would be afraid of reinfection.*
		*I would start PrEP immediately, because I never want to go back to the fear of contracting HIV.*

Motives for participating in cure-related trials		
1. Contributing to new knowledge/science	Beneficial for other people	*.some people have to make this kind of sacrifice and commitment to participate in this kind of research.*
		*Well, as I just said: in the interest of science, I would be willing to take a risk.*
	Reciprocation	*Like I said: because other people participated in research, I can benefit from current medication. You know, that didn't come from nowhere, research participants went through that.*
		*.the benefit is that I can contribute to cure. You do it for others, but on the other hand also a bit for yourself, to feel a little better.*
2. Possibility of personal benefit	Earlier access to new medication	*You contribute to research for a cure. If the treatment is effective, then maybe you can get the treatment quicker. Those can be benefits.*
	Possibility of being cured by the trial	*Imagine that it works (laughs). Then you're lucky. One in a million. Yes.*
		*.of course there is a benefit: what if you're one of the lucky ones that got the cure?*
	Temporarily no more daily medication	*That would be the benefit […] not having to take these pills every day.*

Barriers for participating in cure-related trials		
1. Worry about effects on body	Long-term damage	*.that my body reacts in such a way that it doesn't recover, that it does not go back to normal or that new things will happen to my body that aren't good.*
	Risk of previous ART not working anymore	*Maybe the medication you were on before, will be less effective. The uncertainty of what will happen then, that's what I fear.*
	Risk of ARS coming back	*Getting sick, in the sense of really, really … from what I remember of how I felt at first […] you never want that again.*
2. Practical/financial concerns	Time consuming	*You often invest a lot of time, traveling to the hospital, parking costs, travel costs. You can certainly report these costs, but you should also get some compensation for the effort that you put in.*
	Not being able to work/study	*You have to arrange it at work every time, leaving earlier or coming in later. And what can make it difficult is that maybe you get sick, and you don't know … yes, you have to let your work know. Work is actually the biggest barrier.*
		*.you say: many side effects will look like HIV-symptoms. I think it could affect your work too much and you cannot deal with it. And also: hospital every 2 weeks, that is a very high frequency.*
3. Transmission to partners	No longer care-free, unsafe sex	*I'm going to put other people at risk, so I have to just say: I can't go that far. I would go as far as I can, where I won't harm anyone else, but I couldn't jump in and say: yes.*
	Disclosing status to partner	*I'm not the type to say: okay, I'm HIV positive, but I'm undetectable, so it would be even worse to say: hey, I'm HIV positive, but I'm doing a study.*
4. Becoming detectable	Reminds you of your diagnosis	*I'm so happy that I'm undetectable and I don't have to think about my status.*
	Being undetectable feels secure	*That is awhile back. Yeah, then you are living a bit in uncertainty … I don't know where it is, I don't know what I can and can't do. And that is what I was really focused on in the beginning and what I was really happy to let go of. That then coming back again, that is certainly an issue, let's put it that way.* *That feeling of uncertainty.* *Yeah.*
5. Living with uncertainty	Unknown risks	*I would be a little bit worried, but I hope that you would have a really good discussion about how and what can happen with these things, and what risks they pose. Yeah, of course I would be worried, because you are a bit in the dark. Look, if they can't give enough information, then I don't think so either, say, if it is possible that your body is harmed, and you can't recover, then I wouldn't do it either.*
	Renewed preoccupation with HIV	*But having that uncertainty again that I had in the beginning, that for me would be the worst, because I was really having a difficult time at first.*
		*It wouldn't give me any peace, it would really mess up my inner peace.*

### 3.1 Understanding of HIV cure

The majority of the participants perceived HIV cure as complete elimination of the virus (sterilizing cure), whereas only a few people thought of it as post-treatment control in the absence of ART.

‘.the virus out of my body, for good. Yes, the virus completely out of my body. That would be great, even though I do not feel anything [of living with HIV now], but the idea. Yes’ (Participant 1041).

The knowledge that with current treatment the virus is still present in a reservoir specifically bothered one participant.

‘‘*That it’s out of your body. That it is no longer in a small reservoir with a lid on it, but that even that small piece is no longer detectable. That is cure for me’ (Participant 1005).*

A few participants felt that a cure would be reached when the body can take care of the virus itself, without the need of medication.

‘I think cure is that your own immune system can suppress it, like it does with Pfeiffer [Epstein Barr virus]’ (Participant 1026).

### 3.2 Knowledge of HIV cure-related research

Most participants had limited knowledge of HIV cure-related research. Some of them had heard about cure-related research in the media or in their community. The famous cases of the Berlin patient and the Mississippi baby were mentioned.^18^^,^^19^ Furthermore, some participants mentioned bone marrow transplantation in relation to HIV cure. Some participants did not believe in cure. They considered prevention as the best option; either with vaccination or with pre-exposure prophylaxis (PrEP).

‘It's like they can invent a vaccine to seize the virus. It's only good for the people who are HIV-negative, I think’ (Participant 1034).

When asked if participants imagine cure treatment to be intensive like chemotherapy, a number of participants confirmed this. One participant said he did not imagine it to be intensive, but rather long-term, depending on how long ago a person had been diagnosed. Others imagined it to be easy, like taking a tablet or getting an injection. One participant mentioned the concept of gene-editing.

‘I read that they want to cut in your DNA, at the part where the HIV is. I don't care what I have to do, as long as I can get rid of it. If they have to cut my DNA, so be it’ (Participant 1041).

### 3.3 Importance of HIV cure

More than half of the participants mentioned that a cure would be important to them personally, because it would make them feel relieved or less worried for various reasons. Approximately half of the participants mentioned how the medication was a burden to them in several ways: it confronted them with their health status, and setting alarms and taking the medication at set times could be a logistical challenge, especially when they did not wish to disclose their status to friends or partners. Also, they expressed their worry about the long-term effect of HIV and/or taking ART on their bodies. Most important, however, was the impact of living with a secret and the deep longing to ‘be normal’, and to be no longer be stigmatized. Living with HIV was said to have an enormous social impact.

‘.*It would be such a relief, especially socially. To know there's no more viru. Such a relief. To be able to live without a secret’ (Participant 1022).*

Another participant mentioned the costs of living with HIV for society:

‘I think it is important to cure HIV because in the long term the medical expenses for society are high because of all the medication we [people living with HIV] need in our lives’ (Participant 1022).

In contrast, a number of participants mentioned that being cured from HIV would not be very important to them. Some said they felt to have really grown from the experience of being diagnosed with HIV or they were afraid to go back to their fear of contracting HIV again. For others, a cure was not important, because they did not feel sick:

*‘ … because I'm undetectable and I take a tablet every day I don't feel that I'm cured, but it doesn't weigh on my mind that I have HIV. So if I was to be given a magic tablet that would take it away, I would probably feel the same as I do now’ (Participant 1106)*. Some participants thought living with HIV was not that burdensome, compared to other diseases.

*‘ … if HIV is treated, is it even a disease? Because I do not feel sick at the moment, I do not notice it. I am a lot less sick than people with other conditions. So in a way, I am already cured’ (Participant 1041)*. Similarly, one stated: ‘*.I look at it and think: thank God it is not cancer’ (Participant 1023).*

### 3.4 Knowledge on the relationship between early diagnosis and HIV cure

The majority of participants were not aware of a relationship between treatment of acute HIV infection and cure. Less than half of participants believed their early diagnosis gave them a higher chance of getting cured.

‘The connection with the cure, I don't think so. The only thing is that if you're diagnosed early, you can live a healthy life earlier, maybe’ (Participant 1034).

However, there were also those who made the connection.

‘the earlier you're diagnosed, the less virus is established in reservoirs, the less virus in your blood, the less virus that has to be eliminated’ (Participant 1022).

### 3.5 Joining cure-related trials

Eleven participants would consider participating in scenario 1, which included brief ATI (Box 1).

Several participants mentioned the importance of information in decision-making for both scenarios. *‘I'd want to know all of the information. I wouldn't want anything left out. I don't think that you can make an informed decision if you don't have all the information.’*

… *‘Hmm, I have to think about it. The risk of infecting somebody that's not infected is not a good risk, so. Yes, you'd have to think about it and then you have to change your life to accommodate for [it]. I would think about it, but again, I need all the information’ (Participant 1036).*

Only one participant would agree upon extended ATI as long as this would be physically acceptable. The worries that were expressed by participants for participation in scenario 1, appeared to be deal-breakers for participation in scenario 2.

In scenario 2, most participants worried about staying off ART for an additional month after viral rebound.

‘Scenario 2 seems a lot more intense than scenario 1, because you get less check-ups and as soon as you're detectable, you have to wait for a month … I would say no to this one, out of fear for what happens to your body in the future. What damage is done by walking around with a detectable HIV viral load?’ (Participant 1044).

### 3.6 Motives for participating in HIV cure-related trials

The main motive for participating in HIV cure-related trials was to contribute to science:

‘ … As I said before, I think it is nice to participate in scientific research. It is nice to give back, to be valuable for scientific research and other people’ (Participant 1022).

‘ … “I don't think it's so crazy, also for myself, to make a contribution to something greater. […] yeah, being part of society, making your contribution to society, to something that also benefits me personally. And that can also be in the form of science” (Participant 1016).

A substantial number of participants felt to have a responsibility towards the community to engage in research, because of the sacrifice other people made for them in the past by participating in HIV research.

*“… because other people participated in research, I can benefit from current medication. That did not come from nowhere, research participants went through it [the process of joining a trial]” (Participant 1028).*

Most participants needed more information about the risks and side-effects. Some wished to get very regular check-ups.

Some believed participation could be of personal benefit, either from earlier access to the cure or being cured by the trial itself. Also, a few participants mentioned the advantages of temporarily no ART. Moreover, participants were curious to find out how their body would react to ATI:

‘ … I am really curious: what happens if I stop the medication? Will I be sick in a month, or in a week? I'm curious, and also, I would want to get rid of the pills. That is why I would participate. That, and the future of others' (Participant 1044).

One participant reasoned that he would get priority if a new treatment was found to be effective, because he participated in the (hypothetical) trial. Strikingly, nine out of twenty participants mentioned the possibility of being cured by the trial:

‘Yes, of course there is a benefit: what if you are one of the lucky ones that got the cure?’ (Participant 1023).

‘If participating in a study could give you the possibility of a cure, that would be exciting. If it works, that would be great, it would make me feel better’ (Participant 1022).

### 3.7 Barriers for participating in HIV cure-related trials

Major barriers mentioned were worry about risks of interrupting ART and the consequences of viral rebound. One person mentioned that the research team has to show confidence in the drug, as participants rely on their opinion. Many feared transmitting HIV to their partners after viral rebound, especially in scenario 2:

‘I would not participate, because I am putting my mental and sexual health first now, as well as the sexual health of partners around me. I already worry a lot about these things, so this would be a disproportionate burden for me’ (Participant 1021).

Some participants believed participation would interfere with their work and private life. A few participants referred to the period shortly after their diagnosis as the darkest time of their lives, and initiating ART gave them a sense of security that would be threatened by having to interrupt ART.

### 3.8 Advice to HIV cure researchers

Frequently stated was the wish of the participants to be more engaged in research. They strongly wished to be provided with updates on the status of the NOVA study as well as on current research on HIV cure worldwide. Some participants stated that an explanation of the rationale behind studies would increase their willingness to participate in cure-related trials. Also, several participants mentioned they want researchers to be more transparent about research evidence and possible personal risks and benefits, but also asked of them to be thoughtful of what they share with people living with HIV:

‘Be as transparent as possible and do not create false hope, even when you know about a breakthrough that might be happening, be careful with sharing that’ (Participant 1026).

## 4 Discussion

The present study revealed perceptions of HIV cure and cure-related trials and personal motivations and barriers to participate in cure-related trials among participants in the NOVA study, who had been diagnosed during acute HIV infection and started ART immediately upon diagnosis.

The majority of our participants had limited knowledge about HIV cure and had little conception of what HIV cure would look like. Nearly all viewed HIV cure as a complete elimination of the virus from the body. This would thus correspond with the concept of a sterilizing cure.^20^ Although the IAS recently described a sterilizing cure, in which rebound-competent reservoir is eradicated, as the ‘optimum’ target product profile, more realistic would be a combination of interventions resulting in a sustained period of time in which viral suppression is maintained in the absence of ART (3). Nuances are important in defining HIV cure and we believe this finding emphasizes the importance of being careful with terminology when informing potential candidates about cure-related trials.^20^^,^^21^ As reported previously, the word cure may create misunderstanding and raise unrealistic expectations for potential personal benefit.^20^

The possibility of personal benefit appeared to be an important motive. Many participants said they would be willing to participate out of curiosity about what the effects of study interventions would be on their health. Some mentioned the hope that participation would lead to being cured themselves. It is important to notice that despite having explicitly informed our participants during the interviews that no personal medical benefit from cure-related trial participation was to be expected, almost half of the patients still hoped for it. This finding is consistent with previous studies showing that participants remain hopeful of being cured through trial participation.^13^^,^^22^ This expectation is typical in many early phase trials.^23^ We believe this should be clearly discussed during the informed consent procedures of cure-related trials to ensure that decisions to participate are truly informed.

The main motive behind participation in a cure-related trial was the wish to contribute to science. This was driven by altruistic motives or to reciprocate to researchers and research participants who made current treatment options (i.e. ART) possible through HIV research in the past.

Regardless of our participants’ motives, participants had a sense of the risks and burdens of HIV cure-related research, and as a group were much more comfortable with brief ATI studies, even if some participants would also decline to participate in those. Reasons for not wanting to participate in brief and/or extended ATI ranged from fear of side-effects, practical and financial barriers, to the psychological burden of viral rebound. For most participants, putting sexual partners at risk by participation because of viral rebound was a major concern. Providing accurate information regarding this risk is important in order to remove this concern and guide participants through the study period. When and if viral rebound occurs is dependent on type of intervention, duration of ATI and in which stage of infection ART was initiated.^24^ Nonetheless, extended ATI (and thus perhaps viral rebound) may be necessary to find out if an intervention was truly effective.^4^

Inextricably linked with decision-making in cure-related trial participation is the importance that participants assign to HIV cure. For our participants the social impact of no longer living with HIV was more important than the physical consequences. Participants expressed a strong desire to become “normal”, i.e., not having to worry about setting alarms for medication, disclosure of their HIV status to partners, possible long-term negative effects of HIV and/or ART, and about experiencing HIV stigma. Even though most participants did not experience physical consequences of their HIV infection, almost all would want to be cured if they had a choice. This is in line with the findings of another study performed in the Netherlands, that showed that 82% of Dutch MSM with HIV would be relieved if a cure existed. Predominant reasons were persistent emotional burden caused by HIV status disclosure and stigma, showing the importance of continued efforts in HIV cure-related research.^25^ Furthermore, this also stresses the importance of reducing HIV stigma in society.

However, we observed a small group who expressed a different view and mentioned that they would not instantly opt to be cured, suggesting that not all individuals living with HIV would be willing to participate in a cure-related trial, especially when overall contentment with ART is high. This is in line with findings from a qualitative study among people living with HIV (PLWH) in Guangzhou, China.^26^

There were some remarkable differences between our findings and those in the SEARCH study in Thailand, where participants joined a trial in which an intervention (vorinostat treatment) was followed by ATI.^27^ In contrast to the findings in our study, going off ART was one of the key drivers for participation in the Thai cohort. Thai participants viewed going off ART as a rare opportunity to explore the effects of ATI and also to end the burden of treatment and to feel normal again. The majority of our participants perceived going off ART as stressful and it therefore served as a barrier rather than a driver of participation. Motivations to participate in cure-related research in the Thai cohort were considered to be influenced by the degree of stigma and cultural values.^14^

Interestingly, findings from our study were similar to those from a qualitative study among PLWH in Australia that investigated the willingness to participate in HIV cure-related trials.^11^ Their study included mostly MSM. Participants were recruited through advertising distributed by HIV community organizations. None of the participants had previously participated in HIV cure-related trials, but four had participated in an HIV treatment trial. Similar to our results was the finding that Australian participants viewed ATI as stressful because of the sense of loss of stability and control in the management of their HIV infection. Like in the present study, ‘being undetectable’ was perceived as crucially important for the psychological well-being of the Australian participants.^11^ People who have started ART are well-informed about the importance of adherence, not only for maintaining an undetectable viral load but also for preventing transmission of HIV infection, widely known as the ‘U=U (undetectable = untransmissible)’ slogan. Despite this slogan being launched during the data collection period of the SEARCH-study, SEARCH-study participants were still in favor of going off ART. This is remarkable as most studies have shown that increased viral load causes higher levels of distress or depression compared to when viral load is undetectable, regardless of its impact on physical health.^28^ Being detectable can cause increased stigma and therefore emotional burden.^29^

Receiving (correct) information came forward as a central theme in our interviews. Prior to, but also during study participation. As stated earlier, it was noticed that our participants had some false expectations regarding their chances of personal medical benefit. Interventions have been investigated to improve the informed consent process, like the use of an educational video.^30^^,^^31^ Also, as Peay et al. suggested, integrating decision-making studies and involving social scientists in the informed consent process will help participants make a better informed choice and gives the opportunity for more personalized guidance throughout the cure-related trial.^20^

There are some limitations to be acknowledged in our study. First, our cohort consisted of people diagnosed in the acute phase of HIV infection who started ART immediately thereafter. Their perceptions on HIV cure are not necessarily representative for the larger population of PLWH. However, because they are the most likely candidates for cure-related trials, their perceptions presumably represent those of persons facing the actual decision about joining a cure-related trial. Second, the interviewees were only men, and mostly MSM, so conclusions can only be drawn for this specific group. More efforts are needed to include all key populations in cure-related research. Inherent to being a qualitative study, the number of participants was low. However, thematic saturation was achieved. Finally, our participants were asked to think about a hypothetical scenario of joining a cure-related trial and were not facing an actual decision. Previous research has shown that hypothetical choices and considerations may not always represent actual decisions and considerations.^32^^,^^33^ The undefined time off ART in scenario 2 and the lack of information on the rationale behind this may have influenced participants’ perceptions.

## 5 Conclusion

Based on our results, we propose for the design of future cure-related trials to include involvement of PLWH and social scientists, thorough informed consent procedures and careful use of terminology to ensure that potential participants make truly informed choices. Special attention needs to be paid to HIV transmissibility during ATI, as this emerged as a major concern for most participants.

## Funding

This research was funded by Gilead Sciences, funding number CO-NL-276-422.

## 6 Data statement

Data are available upon reasonable request. We endeavor to make the data used in any The Netherlands Cohort Study on Acute HIV infection (NOVA) manuscript publicly available, within the limits of the ethical governance under which the data were collected. To this end, we will share data directly with interested parties for two purposes: (1) verification and replication of an already published analysis derived from NOVA, (2) novel scientific research projects using NOVA data. To facilitate this, requests for data sharing can be made on a case-by-case basis following submission of a concept sheet. Once submitted the proposed research/analysis will undergo review by the NOVA team for evaluation of the scientific value, relevance to the study, design and feasibility, statistical power and overlap with existing projects. If the proposed analysis is for verification/replication, data will then be made available. If the proposed research is for novel science, upon completion of the review, feedback will be provided to the proposer(s). In some circumstances, a revision of the concept may be requested. If the concept is approved for implementation, a writing group will be established consisting of the proposers (up to three people that were centrally involved in the development of the concept) and members of the NOVA study group (or other appointed cohort representatives). All people involved in the process of reviewing these research concepts are bound by confidentiality. For more information about the procedure, data sharing or collaboration in general, please contact dr. GdB: g.j.debree@amsterdamumc.nl.

## Author's contributions

PvP: conceptualization, coding and data analysis, project management, writing- original draft. MD: conceptualization, data curation, coding & data analysis, methodology, writing-review & editing, tables. SR: conceptualization, data curation, coding & data analysis, methodology, writing-review & editing. HP: conceptualization, writing-review & editing. GH: conceptualization, writing-review & editing. JP: conceptualization, funding acquisition, writing-review & editing. Peter Reiss: writing-review&editing, supervision. CR: writing-review&editing, supervision. AV: writing-review&editing, supervision. PN: conceptualization, data curation, coding & data analysis, methodology, writing-review & editing. GdB: conceptualization, funding acquisition, project management, writing-review & editing.

## Declaration of competing interest

The authors declare the following financial interests/personal relationships which may be considered as potential competing interests.

The authors have no financial or proprietary interests in any material discussed in this article. GdB received honoraria to her institution for scientific advisory board participations for 10.13039/100005564Gilead Sciences and speaker fees from 10.13039/100005564Gilead Sciences. PR received grants from ViiV Healthcare, 10.13039/100005564Gilead Sciences and 10.13039/100004334Merck and participated in a Data Safety Monitoring Board, the honoraria are paid to the institution. CR received grants from Gilead, ViiV, Merck and JJ and payments or honoraria from Gilead and ViiV for viral education.
